# Cutaneous plasmacytoma: a rare manifestation of multiple myeloma^⋆^

**DOI:** 10.1016/j.abd.2022.10.018

**Published:** 2024-02-23

**Authors:** Larissa Helena Marques Carrai, Elaine Cristina Faria Abrahão Machado, Luiza Castro, Livia Matida Gontijo

**Affiliations:** Pontifícia Universidade Católica de Campinas, Campinas, SP, Brazil

Dear Editor,

Cutaneous metastases result from the spread of a tumor to the skin through lymphatic or vascular embolization, direct implantation during surgery, or involvement of the skin through contiguity. Studies indicate a frequency of 0.7%-10.4%, mainly secondary to visceral neoplasms.^1^ The primary neoplasms most often associated with skin metastasis include breast cancer, lung cancer, and melanoma.^2^ There are few reported cases of cutaneous metastasis from multiple myeloma (MM), the main topic in this case report. Skin involvement associated with MM occurs in less than 10% of cases.

Due to the rarity of this manifestation, as well as the importance of its correct diagnosis, the present report describes a patient with MM and cutaneous metastasis after disease recurrence.

A 49-year-old female patient had been diagnosed with MM 12 years before. She underwent several treatments, including a bone marrow transplant. She had a painless lesion on her right leg that had been developing for three months. She had a history of excision of a tumor in the right tibia with prosthetic reconstruction in the previous year. On examination, she had two well-defined, erythematous tumors with regular contours, located on the right pre-tibial region, measuring up to 3 cm (Fig. 1). At the site of the orthopedic prosthesis scar, she had an erythematous, hardened, and painless nodule measuring approximately 2 cm, adhered to deep planes (Fig. 1). The pathological analysis of an incisional biopsy was compatible with a neoplasm of large, poorly differentiated cells, of probable metastatic origin (Fig. 2). Immunohistochemistry was positive for CD79a, CD138 and Kappa, confirming the diagnosis of MM skin metastasis (Fig. 3). In a joint decision with hematology, radiotherapy was chosen due to the poor prognosis. However, the patient moved to another city and was lost to follow-up at the service.

**Figure 1:**
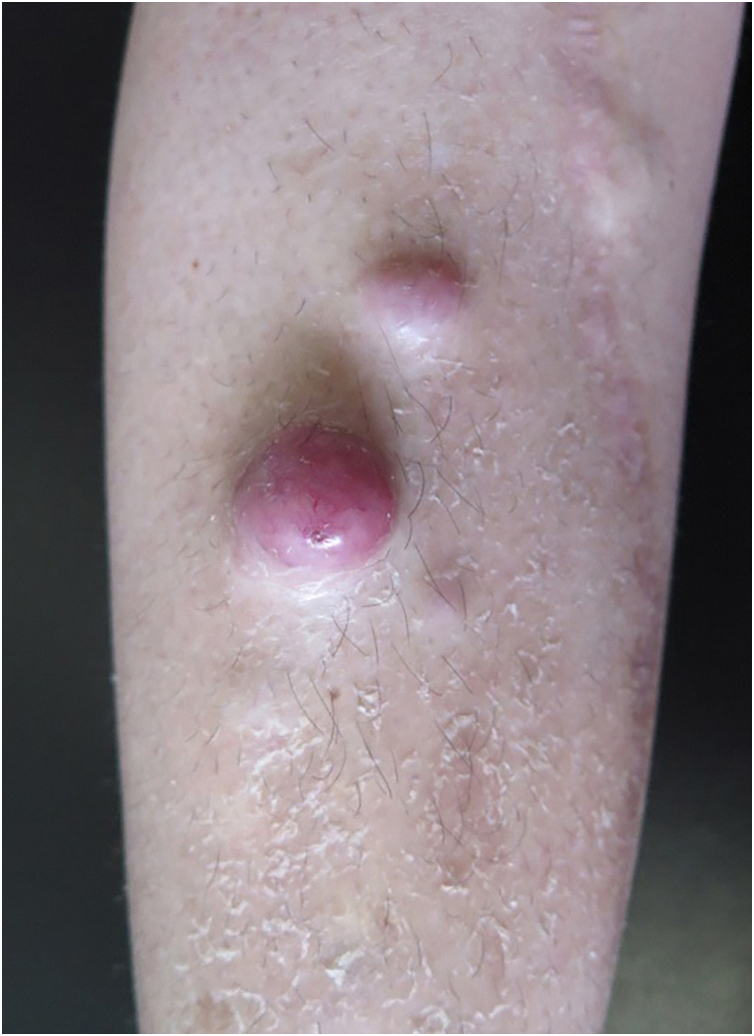
Erythematous tumors on the right pre-tibial region.

**Figure 2:**
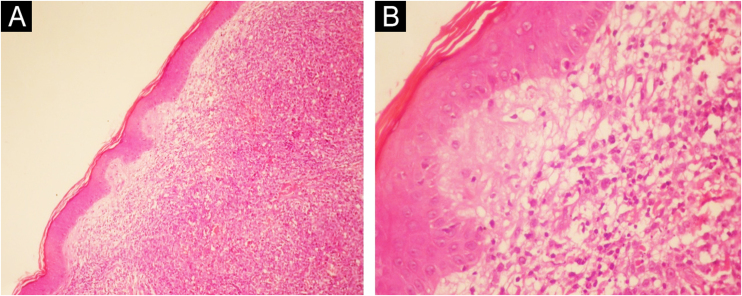
Immunohistochemistry. Diffuse infiltration of the dermis by atypical cells (A); dermal infiltration by large, poorly differentiated cells, suggestive of plasma cells – greater magnification (B).

**Figure 3:**
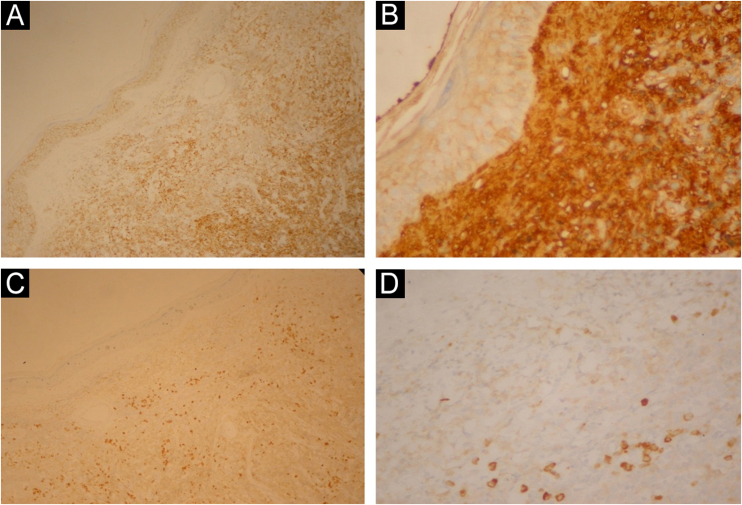
Diffuse positivity for C138 in tumor cells (A); diffuse intense KAPPA positivity in the lesion (B); focal positive CD79A (C); focal positive CD79A – higher magnification (D).

Cutaneous involvement by MM is a rare event, and cutaneous metastasis can appear in any area of the skin, most frequently the trunk, extremities, and face.^3^ Frequently, multiple lesions are observed, although solitary lesions have also been recorded.^4^

Cutaneous metastatic lesions of MM are classified into non-specific ones, which are more common: secondary amyloidosis, alopecia, pyoderma gangrenosum, flat xanthomas, anhidrosis, sclerodermiform lesions, lichen myxedematosus, among others, and specific ones, which represent the spread of multiple myeloma in the final stage of the disease: secondary plasmacytomas that occur by direct extension to the skin from underlying bone lesions, such as in the case described in the present report, or by lymphatic and/or hematogenous spread.^3^, ^4^ They present as erythematous nodules, ulcerated or not, or plaques measuring up to 5 cm in diameter. Around 50% of the patients die within six months of the diagnosis. Cutaneous plasmacytomas can also appear in patients without a previous diagnosis of MM and are then called primary cutaneous plasmacytomas.

Therefore, a thorough dermatological examination is essential for the early diagnosis of cutaneous metastases from multiple myeloma. Thus, it becomes possible not only to optimize patient treatment but also to corroborate the importance of dermatologists because of their responsibility in the diagnosis and follow-up of patients with severe systemic diseases.

## Financial support

None declared.

## Authors’ contributions

Larissa Helena Marques Carrai: Design and planning of the study; drafting and editing of the manuscript; collection, analysis and interpretation of data; critical review of the literature.

Elaine Cristina Faria Abrahão Machado: Design and planning of the study; drafting and editing of the manuscript; collection, analysis and interpretation of data; critical review of the literature.

Livia Matida Gontijo: Approval of the final version of the manuscript; effective participation in research orientation; drafting and editing of the manuscript; collection, analysis and interpretation of data; critical review of the manuscript.

Luiza Castro: Design and planning of the study; drafting and editing of the manuscript; collection, analysis and interpretation of data; critical review of the literature.

## Conflicts of interest

None declared.
